# Proteasome inhibitors restore the STAT1 pathway and enhance the expression of MHC class I on human colon cancer cells

**DOI:** 10.1186/s12929-021-00769-9

**Published:** 2021-11-10

**Authors:** Yi-Hsin Liang, Kuo-Hsing Chen, Jia-Huei Tsai, Yung-Ming Cheng, Chang-Cheng Lee, Chiu-Hwa Kao, Kuang-Yu Chan, Yeh-Ting Chen, Wen-Ling Hsu, Kun-Huei Yeh

**Affiliations:** 1 Graduate Institutes of Oncology, National Taiwan University College of Medicine, No 7, Chung-Shan South Rd, Taipei, 10002 Taiwan, R.O.C.; 2 Graduate Institutes of Clinical Medicine, National Taiwan University College of Medicine, Taipei, Taiwan, R.O.C.; 3 Graduate Institutes of Centers of Genomic and Precision Medicine, National Taiwan University College of Medicine, Taipei, Taiwan, R.O.C.; 4 Departments of Oncology, National Taiwan University Hospital, Taipei, Taiwan, R.O.C.; 5 Departments of Pathology, National Taiwan University Hospital, Taipei, Taiwan, R.O.C.; 6 National Taiwan University Cancer Center, Taipei, Taiwan, R.O.C.

**Keywords:** Colorectal cancer, Major histocompatibility complex (MHC) class I, Proteasome inhibitors, STAT1, Tumor infiltrating lymphocytes (TILs), Interferon-γ (IFN-γ)

## Abstract

**Background:**

A new strategy, particularly a novel combination, for immunotherapy in microsatellite stable metastatic colorectal cancer (mCRC) treatment needs to be formulated. Studies on the interferon-γ (IFN-γ)/ Janus kinase (JAK)/ signal transducer and activator of transcription (STAT)1 pathway provide new directions in this regard.

**Methods:**

Our study applies three colon cancer cell lines, including microsatellite stable (MSS) cell lines, which are SW480 and SW620, and microsatellite instability-high (MSI-H) cell line, which is DLD-1. We compared the expressions of immune surface markers on colon cancer cells in response to IFN-γ. We elucidated these mechanisms, which involved the upregulation of immune surface markers. Furthermore, we examined real-world clinical samples using the PerkinElmer Opal multiplex system and NanoString analysis.

**Results:**

We established that the baseline expression of major histocompatibility complex (MHC) class I alleles and programmed death-ligand 1 (PD-L1) were generally low in cell line models. The immune surface markers were significantly increased after IFN-γ stimulation on SW480 but were notably unresponsive on the SW620 cell line. We discovered that STAT1 and phosphorylated STAT1 (pSTAT1) were downregulated in the SW620 cell line. We verified that the STAT1/pSTAT1 could be restored through the application of proteasome inhibitors, especially bortezomib. The expression of MHC class I as downstream signals of STAT1 was also up-regulated by proteasome inhibitors. The similar results were reproduced in DLD-1 cell line, which was also initially unresponsive to IFN-γ. In real-world samples of patients with mCRC, we found that higher STAT1 expression in tumor cells was strongly indicative of a highly immunogenic microenvironment, with significantly higher expression levels of MHC class I and PD-L1, not only on tumor cells but also on non-tumor cells. Furthermore, tumor infiltrating lymphocytes (TILs) were increased in the positive-STAT1 group. Through NanoString analysis, we confirmed that the mRNA expressions of IFN-γ, human leukocyte antigen (HLA)-A, HLA-E, and HLA-G were also significantly higher in the positive-STAT1 group than those in the negative-STAT1 group.

**Conclusion:**

Our study provides a novel rationale for the addition of bortezomib, a proteasome inhibitor, into new immunotherapy combinations.

**Supplementary Information:**

The online version contains supplementary material available at 10.1186/s12929-021-00769-9.

## Background

The development of immune checkpoint inhibitors has undergone considerable progress for patients with microsatellite instability-high (MSI-H) metastatic colorectal cancer (mCRC), which only comprises 1.8%–4.0% of all mCRC patients [1]. Chemotherapy and targeted therapy remain the primary treatment for more than 96% of patients with mCRC with microsatellite stable (MSS) tumors [2]. Various combination strategies have been established to treat this common form of mCRC; however, two phase III randomized trials provided disappointing results. In the IMblaze 370 study, applying atezolizumab with or without cobimetinib failed to exhibit greater overall survival than applying regorafenib [3]. In the MODUL study cohort-2, maintenance bevacizumab plus 5-fluorouracil (5-FU) with atezolizumab after first-line bevacizumab plus oxaliplatin and 5-FU treatment also failed to exhibit greater progression-free survival than maintenance bevacizumab plus 5-FU alone [4]. Novel strategies, especially combination strategies, for immunotherapy in mCRC with MSS tumors, are crucial for current researches.

Some biomarkers have been identified as predictors for immunotherapy, with the most critical biomarker being the interferon-γ (IFN-γ)/Janus kinase (JAK)/signal transducer and activator of transcription (STAT)1 pathway [5, 6]. The IFN-γ/JAK/STAT1 pathway plays a crucial role in the antigen processing pathway and the subsequent dynamic change of downstream signals, including major histocompatibility complex (MHC) class I [7–12]. STAT1 has also been reported to be a favorable prognostic factor for early-stage colorectal cancer (CRC) [13]. The ratio of STAT1 expression to STAT3 expression is a determinant of CRC growth [14]. In animal models, loss of STAT1 expression promotes the development of CRC [15, 16]. However, the role of the IFN-γ/JAK/STAT1 pathway in mCRC has received little attention [17, 18]. Although IFN-γ also stimulates programmed cell death-ligand 1 (PD-L1) expression, IFN-γ is not a standard treatment for mCRC [19]. Identification of the detailed interactions between STAT1 and PD-L1 might provide new insights for immunotherapy in mCRC.

In previous study, we have focused on the dynamic change of MHC class I in response to chemotherapy agents for mCRC [20]. We demonstrated that the higher expression of MHC class I on colon cancer cells implied higher vulnerability to immune surveillance. In the present study, we attempted to examine the expression of immune surface markers in colon cancer cells and elucidate the mechanisms underlying the effects of STAT1 on immune surface markers. Firstly, we applied our research on MSS colon cancer cell lines, which were SW480 and SW620 [21]. Then we reproduced our results on the MSI-H colon cancer cell line, DLD-1 [21]. Although the correlation between proteasome and colon cancer were studied earlier [22, 23], the detailed interactions between proteasome and STAT1 for mCRC was not addressed before, and thus we would emphasize our research on this mechanism. Further, we also examined the correlations between the IFN-γ/JAK/STAT1 pathway and other clinical characteristics with real-world samples from patients with mCRC.

## Methods

### Cell lines

We employed three colon cancer cell lines, SW480, SW620, and DLD-1 in our study. Both SW480 and SW620 were MSS, while the DLD-1 was MSI-H cell line [21]. All cell lines were purchased from the American Type Culture Collection. The cells were planted in T75 culture flasks (Thermo Fisher Scientific, 156499, Waltham, MA, USA) and maintained in RPMI-1640 medium (Gibco, 31800-022, Waltham, MA, USA) supplemented with 10% FBS (Gibco, 10437-028) and 1% amphotericin B, penicillin, and streptomycin (antibiotic–antimycotic; Gibco, 15240-062) in an atmosphere of 5% CO_2_ at 37 °C.

### Chemicals and other reagents

The compounds applied in this study for colon cancer cell lines were bortezomib (Cayman Chemical, 10008822, Ann Arbor, MI, USA), N-acetyl-L-leucyl-L-leucyl-L-norleucinal (LLnL, Sigma-Aldrich, 208719, Darmstadt, Germany), MG132 (Sigma-Aldrich, 474790), and IFN-γ (R&D Systems, 285-IF-100, Minneapolis, MN, USA).

The chemicals applied for this experiment were xylene (J.T. Baker, JT-9490-03, Radnor, PA, USA), ethanol (Sigma-Aldrich, 32221), formaldehyde solution (10% in aqueous phosphate buffer; Macron Fine Chemicals, H121-08, Radnor, PA, USA), 10× TBS pH 7.4 (Protech, BF204, Taipei, Taiwan, R.O.C), TWEEN 20 (MyBioSource, MBS4156394, San Diego, CA, USA), 10× EDTA buffer pH 8.5 (Sigma-Aldrich, E1161), 10× citrate buffer pH 6.0 (Sigma-Aldrich, C9999), and ProLong Diamond Antifade Mountant (Thermo Fisher Scientific, P36965).

### Flow cytometry

The antibodies applied for flow cytometry were phycoerythrin (PE) anti-human pan-MHC class I (BioLegend, 311406, San Diego, CA, USA), purified anti-human HLA-A (HLA-A; MyBioSource, MBS438658), PE anti-mouse immunoglobulin G1 (IgG1; BioLegend 406608), purified anti-human HLA-C (HLA-C; BioLegend, 373302), PE anti-mouse IgG2b (IgG2b; BioLegend, 406708), PE anti-human HLA-E (HLA-E; BioLegend, 342604), PE anti-human HLA-F (HLA-F; BioLegend, 373204), PE anti-human HLA-G (HLA-G; BioLegend, 335906), PE anti-human CD274 (B7-H1, PD-L1, BioLegend, 329706), PE-IgG1 isotype control (BioLegend, 400112), PE IgG2a isotype control (BioLegend, 400212), and PE-IgG2b isotype control (BioLegend, 402204).

The flow cytometry procedures were described in the earlier study [20]. Briefly summarized, we seeded 10^6^ cells with 10-mL culture medium in 10-cm dishes for 24 h. These cells were treated with IFN-γ 100 U/mL for the subsequent 24 h and then were harvested with trypsin (Gibco, 15400-054) and centrifuged with 500 g for 5 min. In each eppendorf tube, 5 × 10^5^ cells were infused with 200 μL cold phosphate-buffered saline (PBS; Gibco, 10010-023) and recentrifuged. After removing the supernatant, 5 μL of fluorescent antibody (including isotype control antibodies) with 50 μL cold PBS (antibody: cold PBS = 1:10) were applied and the samples were put on ice and in darkness for 20 min. After centrifuging with 500 g for 5 min for twice, the cells were analyzed by flow cytometer (BD FACSCalibur; BD Biosciences, Franklin Lakes, NJ, USA).

### Western blot

We applied enhanced chemiluminescence Immobilon Crescendo Western horseradish peroxidase (HRP) substrate (Merck Millipore, WBLUR0500, Darmstadt, Germany) for these Western blots. The antibodies applied for Western blots were STAT1 (Cell Signaling Technology, #9172, Danvers, MA, USA), phosphorylated STAT1 (pSTAT1; Cell Signaling Technology, #9167), phospho-JAK1 (pJAK1; Cell Signaling Technology, #3331), JAK1 (Cell Signaling Technology, #3332), phospho-JAK2 (pJAK2; Cell Signaling Technology, #3771), JAK2 (Cell Signaling Technology, #3230), interferon regulatory factor-1 (IRF-1; Cell Signaling Technology, #8478), pan-MHC class I (Origene, AM33035PU-N, Rockville, MD, USA), ß-actin (Abcam, ab8227, Cambridge, UK), GAPDH (Abcam, ab8245), HRP donkey anti-rabbit IgG (BioLegend, 406401), and HRP goat anti-mouse IgG (BioLegend, 405306).

The Western blot procedures were described amply in the earlier study [20]. Briefly summarized, protein concentrations were measured using the bicinchoninic acid assay. Then the electrophoresis was applied in 10% sodium dodecyl sulfate polyacrylamide gel electrophoresis (SDS-PAGE, 15 µg per lane) and transferred onto polyvinylidene fluoride blotting membranes (Merck Millipore, IPVH00010). The membranes were incubated in 5% nonfat milk in TBS containing 0.1% TWEEN 20 for blockage of nonspecific binding sites for 1 h, and the membranes were incubated overnight at 4 °C with primary antibodies. On the next day, the membranes were washed with TBS containing 0.1% TWEEN 20 before and after incubation with HRP-conjugated secondary antibodies. These membranes were then incubated with enhanced chemiluminescence Western blotting detection reagents (Merck Millipore, WBLUR0500, Darmstadt, Germany) and exposed to film.

### Patients and clinical samples

We enrolled patients at National Taiwan University Hospital with the following eligibility criteria: (1) patients aged ≥ 20 years; (2) patients with metastatic CRC; and (3) patients without known history of receiving immune modification drugs, such as steroids, immunosuppressive agents, and immunotherapy under clinical trial settings. Informed consents were obtained at diagnosis of metastatic disease status. Primary tumor tissues, including archival tissues or fresh biopsies, from these participants were obtained for subsequent analysis after receiving informed consents.

### Immunohistochemical staining of patient samples

The IHC staining procedures were detailed in the earlier study [20]. Briefly summarized, we applied the Opal 7 Solid Tumor Immunology Kit (PerkinElmer, OP7TL4001KT) for multiplex IHC staining. The PerkinElmer Opal multiplex system could simultaneously apply up to 7 different IHC staining for each cell. The 4′,6-diamidino-2-phenylindole (DAPI) was applied for identification of nucleus. The pan-cytokeratin (pan-CK) was applied for identification of CRC cells. The rest of five biomarkers were applied for identification of immune microenvironment. The antibodies applied for immunohistochemical (IHC) staining were STAT1 (Abcam, ab31369), PD-L1 (Cell Signaling Technology, #13684), MHC class I (Abcam, ab134189), CD4 (PerkinElmer, OP7TL4001KT), CD8 (PerkinElmer, OP7TL4001KT), and pan-CK (Abcam, ab7753). We applied EDTA buffer pH 8.5 (Sigma-Aldrich, E1161), citrate buffer pH6.0 (Sigma-Aldrich, C9999), antibody diluent (PerkinElmer, ARD1001EA, Waltham, MA, USA), Opal Polymer HRP secondary antibody solution (PerkinElmer, ARH1001EA), Opal fluorophore solution (PerkinElmer, FP1487001KT), DAPI solution (PerkinElmer, FP1490A), and ProLong Diamond Antifade Mountant (Thermo Fisher Scientific, P36965) in our study. Quantification of the biomarkers in the tissue sections were performed using the Vectra Polaris Automated Quantitative Pathology Imaging System along with inForm analysis software (PerkinElmer, CLS143455). Detailed information is available on the official website at https://www.perkinelmer.com/lab-solutions/resources/docs/DTS_1-05-40-NR-OPALGUIDELINES_Opal4-7-color_Manual_Kit_Insert.pdf

### NanoString assay of patient samples

We applied a commercial multiplexed gene expression panel, the NanoString nCounter PanCancer Immune Profiling Panel (NanoString Technologies, Seattle, WA, USA). The NanoString panel can simultaneously quantitate 770 immune-related genes through mRNA quantification. All procedures, including preparation, hybridization, detection, scanning, and normalization, were performed according to the manufacturer’s instructions. Detailed information and the gene list are available on the official website at https://www.nanostring.com/products/gene-expression-panels/gene-expression-panels-overview/hallmarks-cancer-gene-expression-panel-collection/pancancer-immune-profiling-panel. Briefly summarized, we obtained 12–15 slides of 10 μm-thickness from formalin-fixed paraffin-embedded specimens. After referring to the representative hematoxylin and eosin slide, viable tumor portion was microdissected from the other unstained slides. Total 200–500 ng RNA was extracted and the percentage of RNA fragments greater than 300 nucleotides (DV300) was greater than 20%. After hybridization with the Immune Profiling Panel by the NanoString Prep Station (NanoString Technologies), sample analysis was performed on a nCounter Digital Analyzer (NanoString Technologies). The raw data processing, quality control, and normalization were performed using the nSolver 4.0 analysis software (NanoString Technologies) after reference gene normalization with 40 house-keeping genes.

### Statistical analysis

All results, except for clinical samples, are based on at least three independent tests. The results are presented in terms of the mean with standard deviation, and are graphed. We applied ImageJ (RRID:SCR_003070, National Institutes of Health, Bethesda, MD, USA) to quantify the results of Western blots. We performed a paired samples *t* test or ANOVA as indicated. All *p* values were two-tailed. The *p* values are marked with asterisks to indicate their level of significance. All data analyses were performed using SPSS 20.0 (RRID:SCR_002865, IBM, Armonk, NY, USA).

## Results

**Fig. 1:**
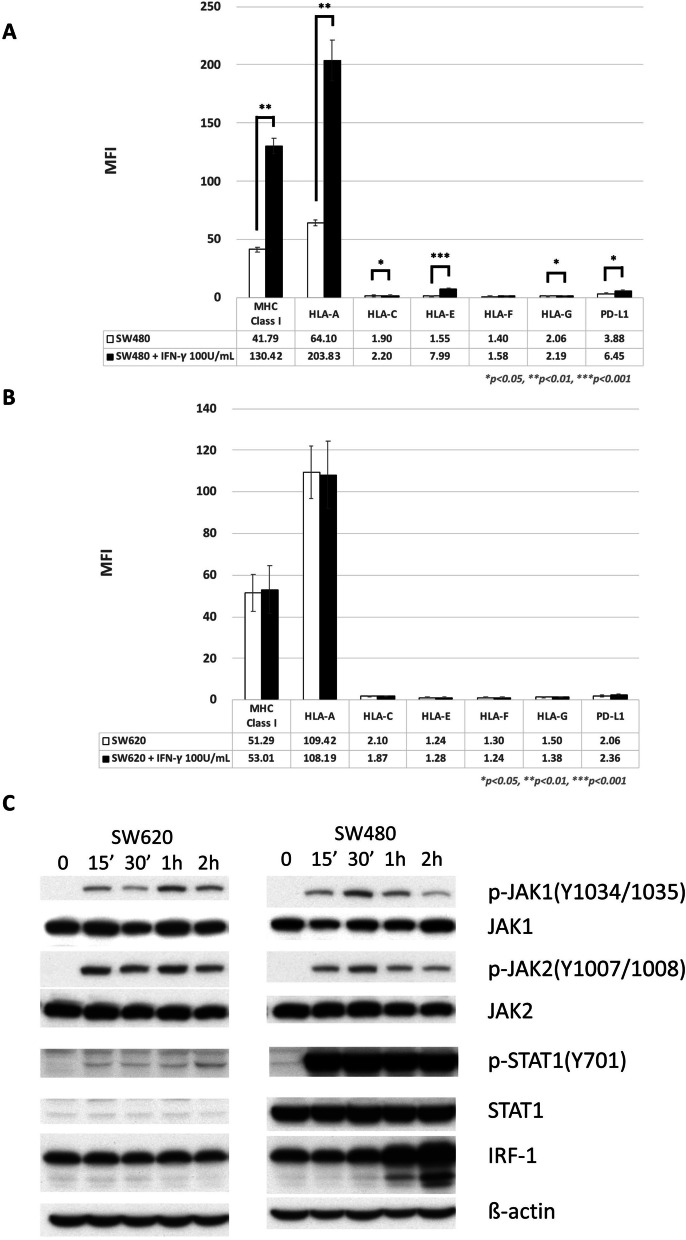
Flow cytometry of SW480 (**A**) and SW620 (**B**) for MHC class I alleles and PD-L1 expressed in response to IFN-γ after 24-h exposure. **C** Western blots for the SW480 and SW620 cells in the IFN-γ pathway in response to IFN-γ after 15 min (15’), 30 min (30’), 1 h (1H), and 2 h (2H). Mean fluorescence intensity (MFI)

*IFN-γ specifically stimulated the expression of stimulatory MHC class I alleles and PD-L1 in SW480 but not in SW620 cells.* First, we examined two colon cancer cell lines, SW480 and SW620, through flow cytometry. The SW480 line demonstrated low expression of all MHC class I alleles and PD-L1 at baseline (Fig. 1A). IFN-γ significantly stimulated the expression of PD-L1 and MHC class I alleles except for HLA-F. In contrast, the SW620 line was completely unresponsive to IFN-γ stimulation for all MHC class I alleles and PD-L1 (Fig. 1B). To elucidate the differences between the SW480 and SW620 cell lines, we subsequently investigated the IFN-γ pathway by using Western blot. After exposure to IFN-γ, the downstream signals of JAK1/JAK2, STAT1, and IRF1 increased in SW480 cells (Fig. 1C). By contrast, STAT1 and pSTAT1 were downregulated in the SW620 cell line.

**Fig. 2:**
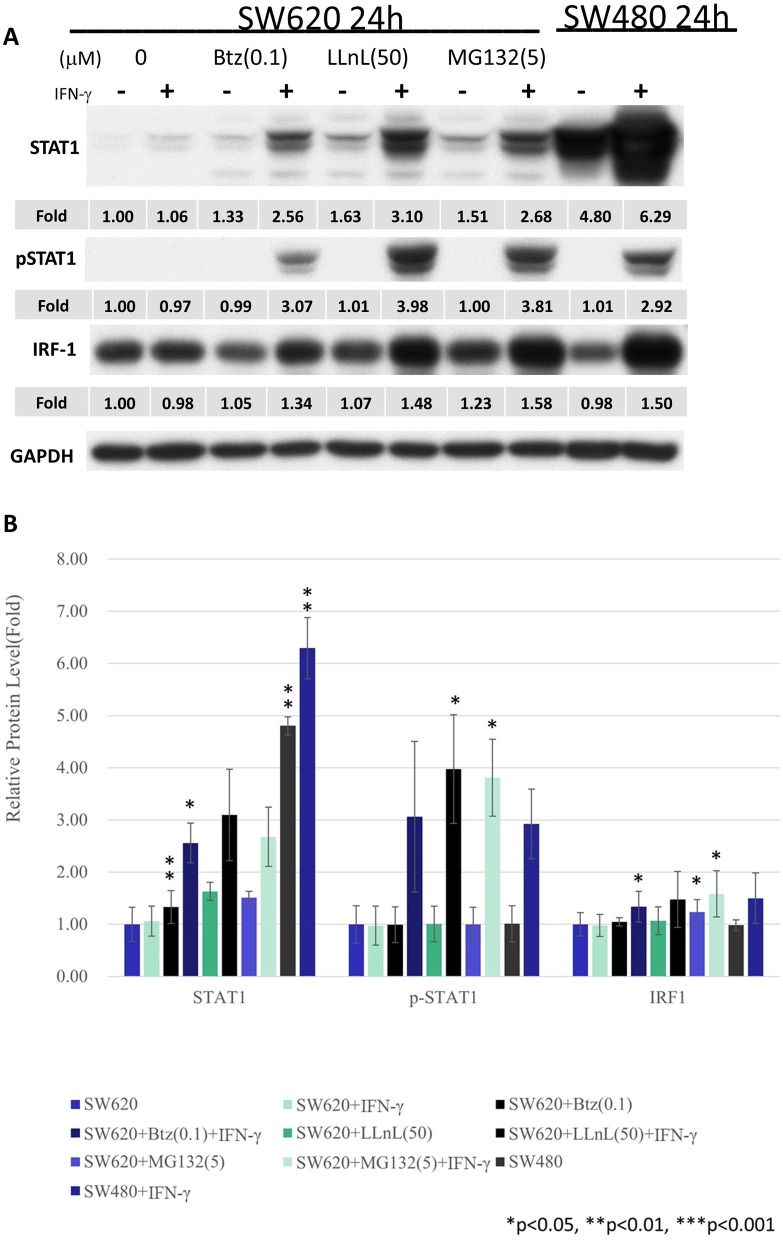
Western blots (**A**) and the quantification of Western blots (**B**) for SW620 and SW480 cells in the STAT1 pathway in response to bortezomib, LLnL, MG132, and IFN-γ

*Proteasome inhibitors restored the expression of pSTAT1 and downstream signals, including IRF1 and MHC class I in SW620 cell line.* The proteasome–ubiquitin mechanism is crucial for the modulation of immune systems [24]. Thus, we examined three proteasome inhibitors, namely bortezomib, LLnL, and MG132. All three proteasome inhibitors restored the levels of STAT1 and pSTAT1. The downstream signal of IRF1 was also upregulated (Fig. 2A & B).

**Fig. 3:**
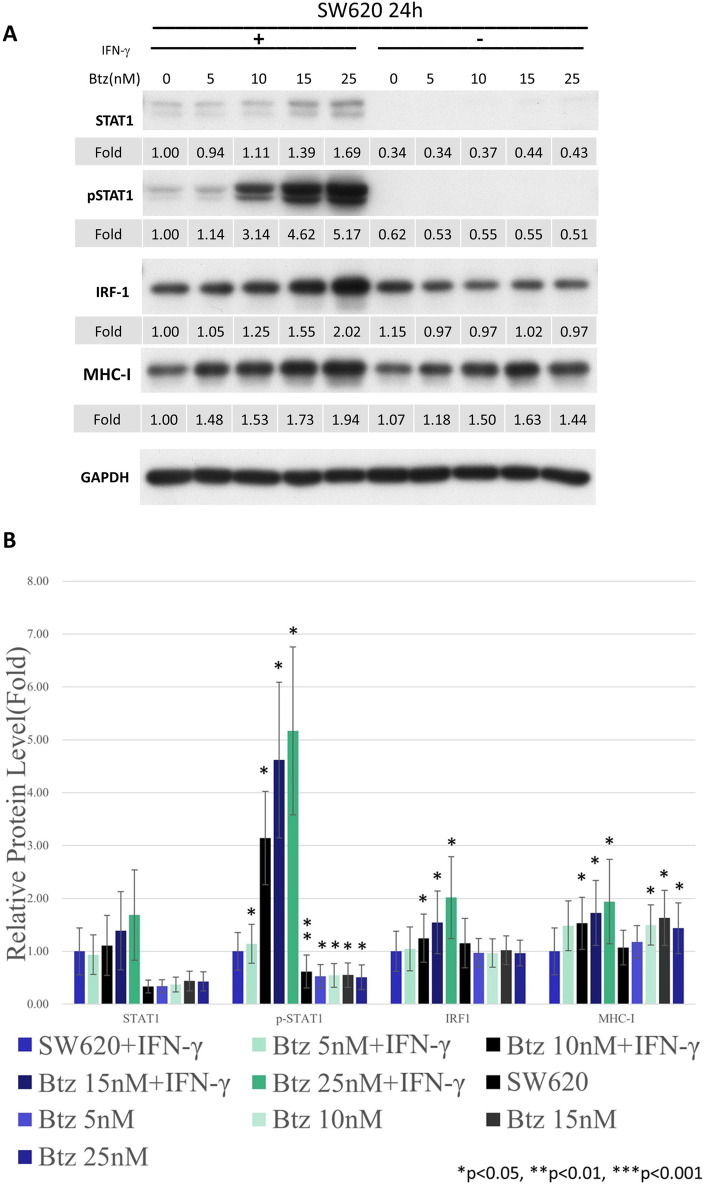

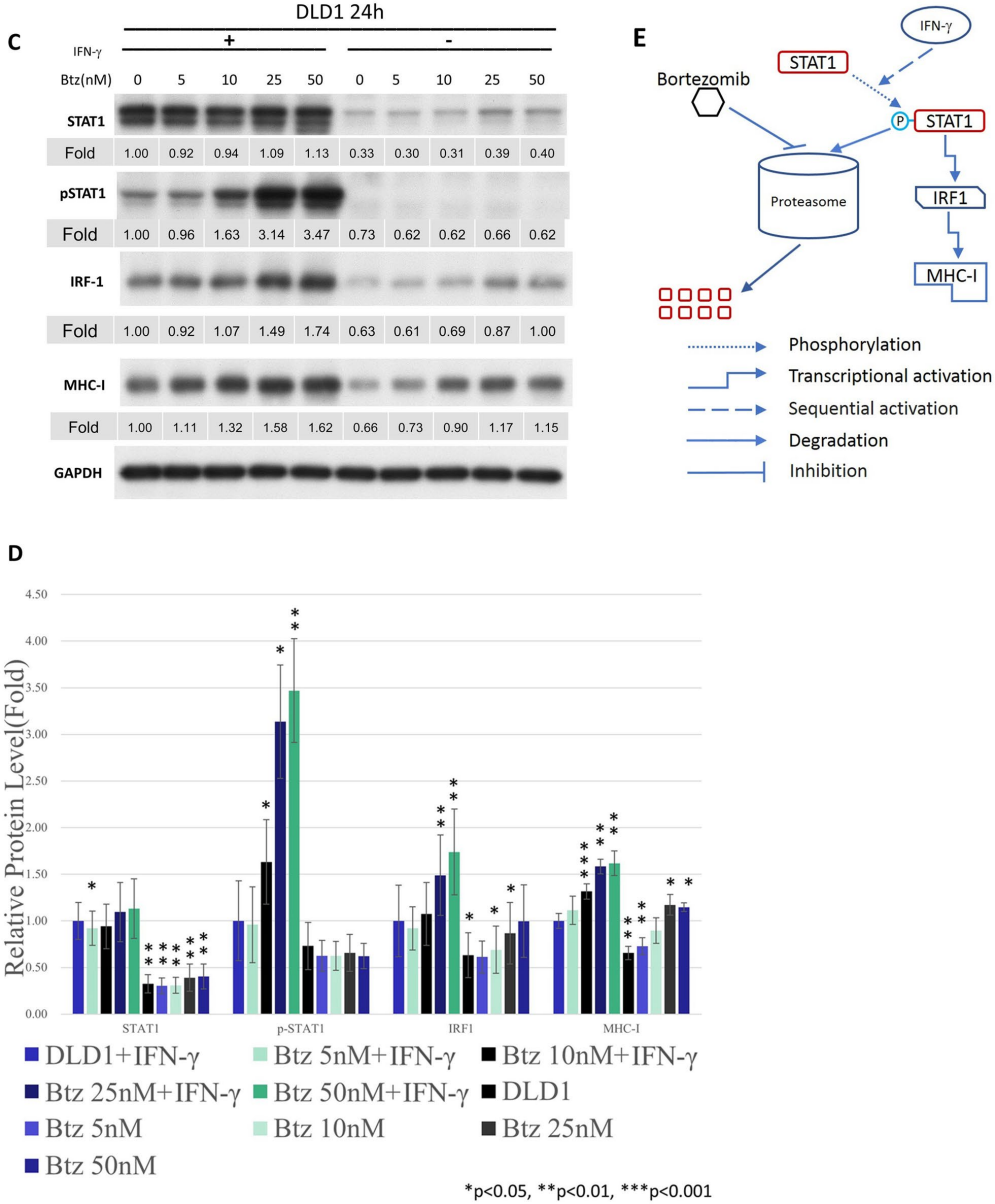
Western blots (**A**) and the quantification of western blots (**B**) for SW620 cells in the STAT1 pathway in response to bortezomib and IFN-γ. We further applied DLD-1 cell line with western blots (**C**) and the quantification of western blots (**D**) for confirmation. Schema of the mechanism of proteasome inhibitors on MHC class I expression of colon cancer cell lines was summarized in Fig. 3 (**E**)

*Bortezomib enhanced MHC class I expression in a dose-dependent manner in SW620 and DLD-1 cell lines.* Because bortezomib is currently one of the standard treatments for hematological malignancies, we further examined its dosage efficacy in SW620 cell line [25, 26]. The levels of pSTAT1 and IRF1 increased with the dosage of bortezomib. The expression of MHC class I was stimulated by the application of bortezomib in a dose-dependent manner (Fig. 3A & B). To reproduce these results, we applied another cell line in our study. As shown in the Fig. 3C and D, the levels of pSTAT1 and IRF1 were restored with the dosage of bortezomib in the DLD-1 cell line. Again, the expression of MHC class I was also stimulated by the application of bortezomib in the DLD-1 cell line. In summary, proteasome might degrade STAT1 at baseline and thus tumor cells were unresponsive to IFN-γ stimulation. The proteasome inhibitors restore the expression of STAT1 in SW620 and DLD-1 cell lines. The stimulation of IFN-γ/JAK/STAT1 pathway subsequently upregulated the expressions of MHC class I (Fig. 3E).

**Fig. 4:**
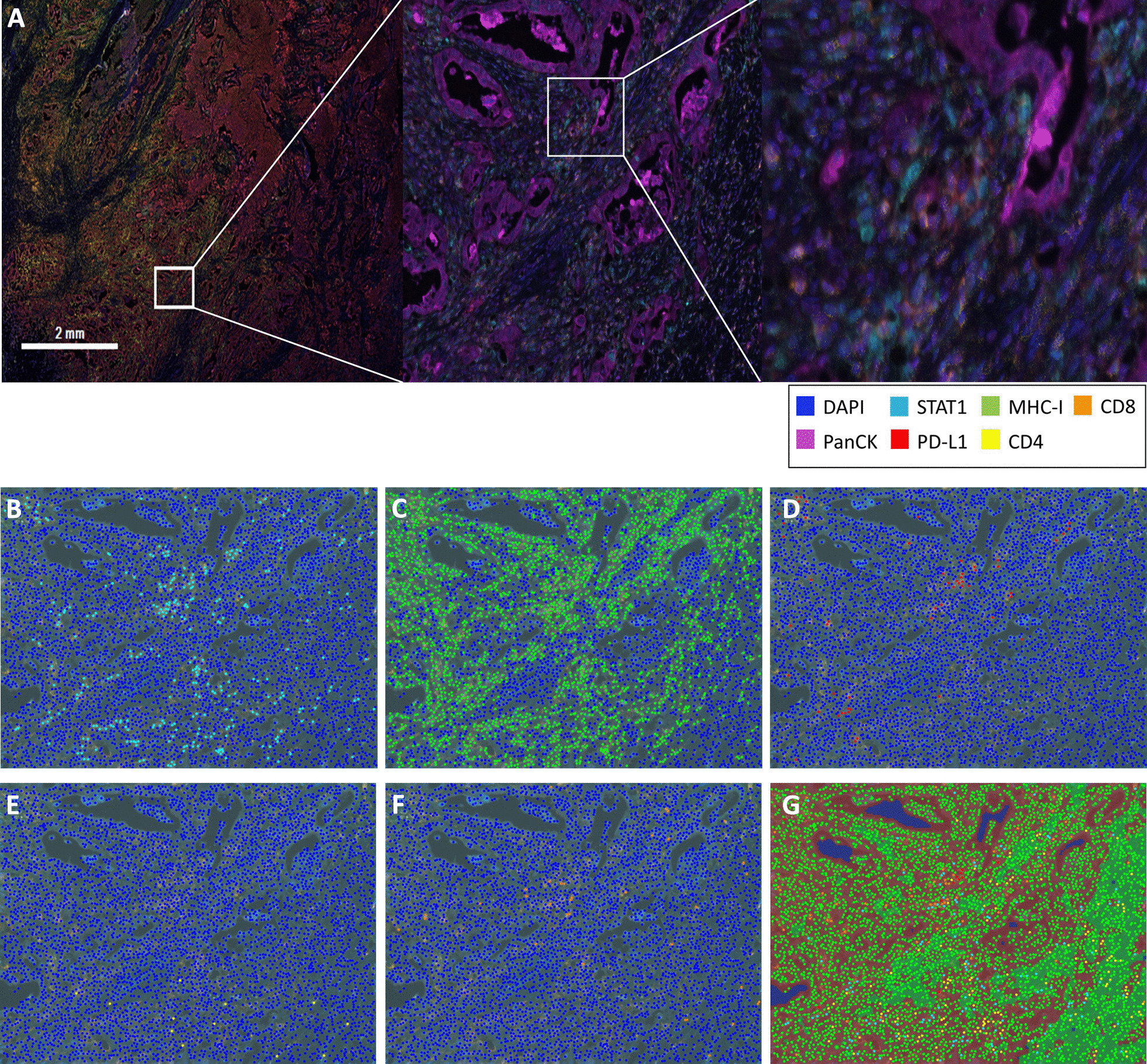
One example of IHC staining by the PerkinElmer Opal multiplex system. The merge of all seven different IHC staining within one picture was demonstrated as (**A**). The individual computerized scanning of STAT1 IHC staining (**B**), MHC class I IHC staining (**C**), PD-L1 IHC staining (**D**), CD4 IHC staining (**E**), and CD8 IHC staining (**F**) were demonstrated. Computerized scanning of superimposing all five IHC staining was demonstrated in (**G**)

*MHC class I and PD-L1 expression were strongly correlated with STAT1 expression in real-world patients’ samples.* We enrolled 60 patients with mCRC in this study. Their clinical characteristics and molecular biomarkers are summarized in Table 1. Only 4 patients had MSI-H tumors and the rest of 56 patients had MSS tumors. All 60 tumor tissues from primary colorectal tumors before mCRC treatment were examined with the PerkinElmer Opal multiplex system, which combines seven different IHC staining signals for each cell within one image, as shown in Fig. 4A. The immune microenvironment was composed by tumor cells and peri-tumor non-tumor cells, including immune cells. As defined by previous studies, tumor cells were identified as both pan-CK-positive and DAPI-positive cells. Non-tumor cells infiltrated in the tumors, such as tumor infiltrating lymphocytes (TILs), were categorized as pan-CK negative and DAPI-positive cells [27, 28]. We applied another 5 biomarkers, which were STAT1, CD4, CD8, MHC class I, and PD-L1, in reference with DAPI and CK to construct the immune microenvironment for each sample. Because the raw image data was too complicated to be visualized manually, we applied computerized scanning of each IHC staining for all 60 samples, and only demonstrated one patient sample as an example in Fig. 4B–G. The computerized scanning processed the STAT1 (Fig. 4B), MHC class I (Fig. 4C), PD-L1 (Fig. 4D), CD4 (Fig. 4E), CD8 (Fig. 4F) respectively. These signals were combined in reference of DAPI and CK to identify the interaction of tumor cells and non-tumor cells by computer (Fig. 4G). As shown in Fig. 4G, the tumor parts were marked as brown background and the non-tumor parts were marked as green background. For example, we could identify the STAT1 expression and the MHC class I expression on each tumor cell and/or non-tumor cell in reference of the synchronous expression of DAPI and CK or not. These statistic results were summarized in Table 1 and more detailed results could be found in the Additional file 1. The definitions of positivity for these biomarkers were referenced by the definition of PD-L1. The definitions of positivity of STAT1, PD-L1, CD4, and CD8 were defined as positive expression on ≧ 1% of tumor cells or non-tumor cells, respectively. The definition of positivity of MHC class I was defined as positive expression on ≧ 50% of tumor cells or non-tumor cells. We observed that 32 (53.3%) of 60 samples were STAT1 positive tumors. STAT1 expression was not correlated with other clinical factors, such as RAS and MSI-H status. However, a positive tumor STAT1 expression implied a strong immune microenvironment. The positive tumor STAT1 expression was significantly associated with higher PD-L1 expression and higher MHC class I expression, on tumor cells and also non-tumor cells. The level of TILs also increased in the positive STAT1 group with significantly more cells expressed CD4 or CD8.

**Table 1:** Patient clinical characteristics

Characteristic	All patients (%)^a^	Tumor STAT1 ≧ 1% (%)^a^	Tumor STAT1 < 1% (%)^a^	*p*-value
All	60 (100%)	32 (53.3%)^b^	28 (46.7%)^b^	
Gender				
Female	31 (51.7%)	16 (50.0%)	15 (53.6%)	0.782
Male	29 (48.3%)	16 (50.0%)	13 (46.4%)	
Age (years)				
Median (Range)	59.8 (36–84)	54.6 (38–85)	62.5 (36–81)	
Age ≤ 65 years	42 (70.0%)	23 (71.9%)	19 (67.9%)	0.735
Age > 65 years	18 (30.0%)	9 (28.1%)	9 (32.1%)	
Primary site				
Right-sided^c^	22 (36.7%)	11 (34.4%)	11 (39.3%)	0.694
Left-sided^d^	38 (63.3%)	21 (65.6%)	17 (60.7%)	
Initial stage				
I–III	4 (6.7%)	4 (12.5%)	0 (0%)	0.156
IV	56 (93.3%)	28 (87.5%)	28 (100%)	
Mutation status				
Wild type	26 (43.3%)	16 (50%)	10 (35.7%)	0.516
RAS mutant	25 (41.7%)	12 (37.5%)	13 (46.4%)	
BRAF mutant	6 (10.0%)	3 (9.4%)	3 (10.7%)	
MSI-H	4 (6.7%)	1 (3.1%)	3 (10.7%)	
Tumor PD-L1				
< 1%	27 (45.0%)	7 (21.9%)	20 (71.4%)	< 0.001^e^
≧1%	33 (55.0%)	25 (78.1%)	8 (28.6%)	
Non-tumor PD-L1				
< 1%	48 (80.0%)	22 (68.8%)	26 (92.9%)	0.020^e^
≧1%	12 (20.0%)	10 (31.2%)	2 (7.1%)	
Tumor MHC-1				
< 50%	12 (20.0%)	0 (0.0%)	12 (42.9%)	< 0.001^e^
≧50%	48 (80.0%)	32 (100.0%)	16 (57.1%)	
Non-tumor MHC-1				
< 50%	20 (33.3%)	6 (18.7%)	14 (50.0%)	0.010^e^
≧50%	40 (66.7%)	26 (81.3%)	14 (50.0%)	
Non-tumor CD4, CD8				
Both < 1% CD4 or CD8 ≧1%	34 (56.7%) 26 (43.3%)	11 (34.4%) 21 (65.6%)	23 (82.1%) 5 (17.9%)	< 0.001^e^

^a^Percentage from subgroup analysis. ^b^Percentage is based on all 60 cases. ^c^Including the cecum, ascending colon, and transverse colon. ^d^Including the splenic flexure, descending colon, sigmoid colon, and rectum. ^e^*p* < 0.05

**Fig. 5:**
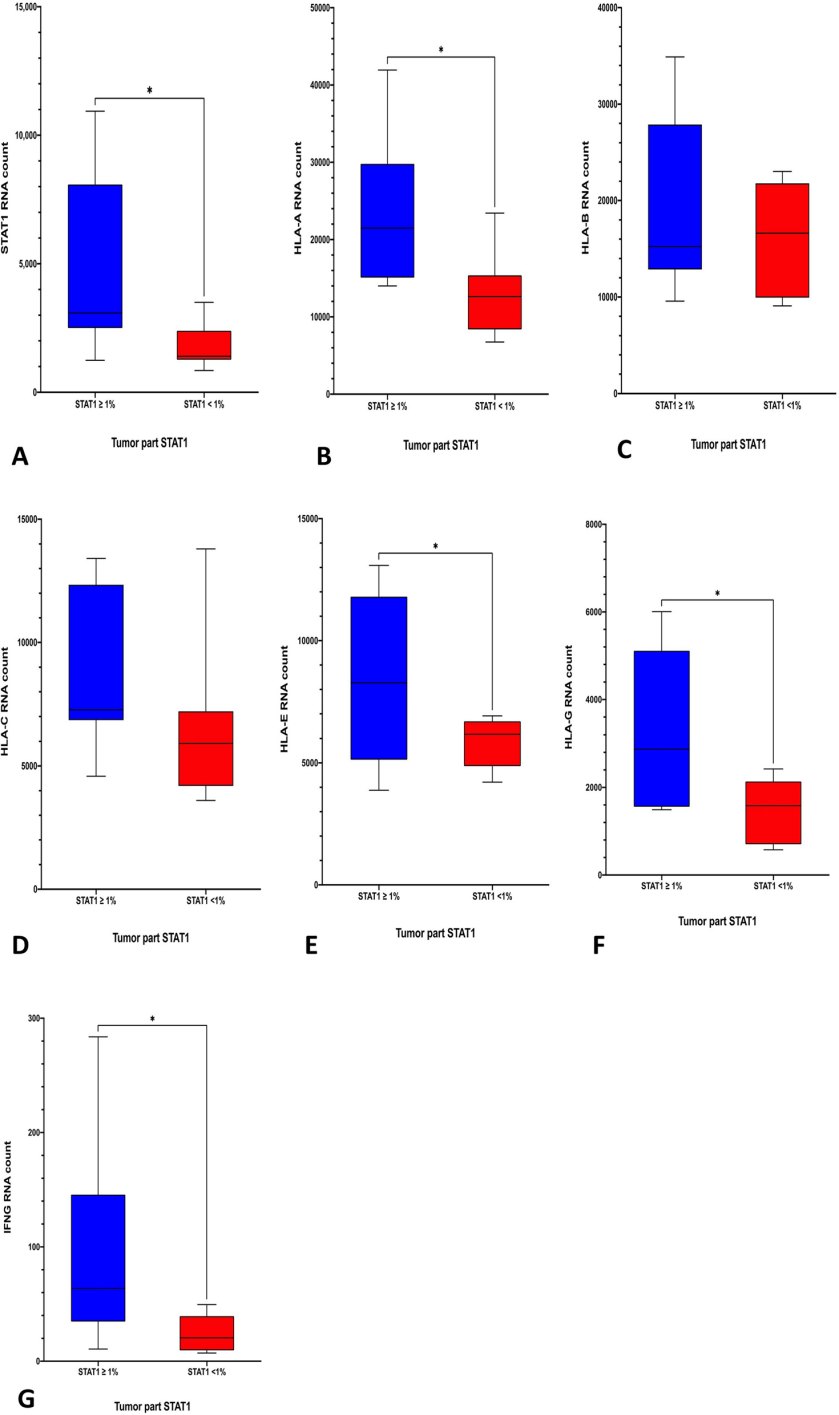
Counts for mRNA expression in tumor tissue through NanoString analysis for STAT1(**A**), HLA-A (**B**), HLA-B (**C**), HLA-C (**D**), HLA-E (**E**), HLA-G (**F**), and IFN-γ (**G**). Blue bar indicates the results of the high-STAT1 group in the Table 1. Red bar indicates the results of the low-STAT1 group in Table 1. *p < 0.05

*NanoString analysis indicated that MHC class I expression, especially that for HLA-A, and IFN-γ*, *significantly increased in real-world patients’ samples.* We further subjected these patients’ tumors samples to NanoString analysis. Consistent with the grouping of results from the PerkinElmer Opal multiplex system, STAT1 expression was significantly higher in the positive-STAT1 group than in the negative-STAT1 group (Fig. 5A). The expressions of all specific MHC class I alleles were higher in the positive-STAT1 group, and the differences were especially significant for HLA-A, HLA-E, and HLA-G (Fig. 5B–F). The NanoString analysis also verified the reason that high STAT1 and MHC class I expression was strongly correlated with IFN-γ expression. The IFN-γ expression was significantly higher in the positive-STAT1 group than in the negative-STAT1 group (Fig. 5G).

## Discussion

In this study, we demonstrated that colon cancer can be separated into two groups according to their response or lack of response to IFN-γ. We discovered that SW620 was irresponsive to IFN-γ because of the downregulation of STAT1. We further found that bortezomib, a proteasome inhibitor, can restore STAT1 expression and subsequently enhance the downstream expression of MHC class I. The real-world mCRC samples were classified into two groups according to their STAT1 expression level. The positive STAT1 expression in mCRC cells was strongly indicative of a highly immunogenic microenvironment, with significantly higher expression levels of MHC class I and PD-L1, in both tumor and nontumor parts, compared with cells with negative STAT1 expression. STAT1 expression was an independent biomarker without any interaction with other molecular biomarkers, such as RAS or MSI-H mutations.

Currently, microsatellite stable mCRC is still the no man’s land for immunotherapy. In previous study, we have demonstrated that the up-regulation of MHC class I made the colon cancer cells more vulnerable to immune surveillance [20]. In this study, we confirmed that the positive tumor STAT1 expression implied higher MHC class I expression and PD-L1 expression on tumor cells and also peri-tumor non-tumor cells, such as TILs. STAT1 expression levels of mCRC might be a predictive biomarker for novel immunotherapy. Furthermore, we have established that bortezomib is a candidate of immunotherapy combination for the negative STAT1 expression mCRC.

Although our research indicated that the STAT1 pathway was independent to microsatellite status and could be applied for mCRC with either MSS tumors or MSI-H tumors, the impactful value of our results was addressed on the novel immunotherapy strategy for MSS mCRC. As mentioned above, the MSI-H mCRC comprised only small proportions of the whole mCRC and current immunotherapy provided perfect efficacy on MSI-H mCRC. On the contrary, MSS mCRC was resistant to immunotherapy. The STAT1 pathway provided a new biomarker and a potential mechanism of action for immunotherapy on MSS mCRC.

STAT1 plays a crucial role in determining the immune microenvironment in mCRC. STAT1 has a strong interaction with indoleamine-2,3-dioxygenase-1 expression in Paneth cells and with subsequent immune escape in CRC [29]. STAT1 play an important role in early tumorigenesis of CRC [30, 31]. Sakahara et al. demonstrated that the STAT1 might be correlated with the drug response [16]. Furthermore, IFN‑γ/JAK/STAT1 signaling stimulates PD‑L1 expression in CRC cells [32]. Kikuchi et al. have indicated that the co-expression of pSTAT1 and PD-L1 in CRC cells was strongly correlated with CD4- and CD8-positive TILs [33]. Our present study discovered the strong correlation with the IFN‑γ/JAK/STAT1 signaling and the major immunogenic action from the stimulation of MHC class I. By using PerkinElmer Opal multiplex system, we drew the same results that the higher expressions of STAT1 and MHC class I subsequently enhanced “hot” immunogenic microenvironment, which was recognized by significantly higher percentage of TILs. The upregulation of STAT1 implied a “hot” immunogenic microenvironment, which might indicate greater vulnerability to immunotherapy.

Several uncertainties remain regarding immunotherapy for microsatellite-stable mCRC. Various combination strategies, including the incorporation of standard chemotherapy or novel targeted therapy, have failed to deliver satisfactory results. A new pathway or novel combination is crucial for effective treatment. The proteasome–ubiquitin pathway plays a major role in regulating STAT expression and thus represents a potential breakthrough [24]. Our research indicated that bortezomib, a proteasome inhibitor, restored MHC class I expression, and thus subsequently transformed the “cold” immune microenvironment into a “hot” microenvironment. Proteasome inhibitors are favorable candidates for further immunotherapy combinations.

The present study has some limitations. First, our data could be applied on STAT1-related mCRC but not all mCRC subtypes. mCRC is a heterogeneous entity that has been classified into at least four specific molecular subtypes [34, 35]. More novel treatments are required for effective treatment in various immune microenvironments. Second, the stimulation of MHC class I might only be the first step in effective treatment. Our study simplified the role of a single agent, and the complexity of interactions for heterogeneous combinations requires further research. Third, we also require another validation cohort comprising more distinct clinical factors among samples to confirm our results. Finally, clinical trials are required to verify our results.

Our study successfully established a mechanism underlying immune escape in mCRC through STAT1 downregulation. We also developed an intervention with a proteasome inhibitor to restore the expressions of STAT1 and MHC class I, which is most critical. Our research provides a completely novel rationale for further immunotherapy combinations. This may represent a breakthrough in addressing the challenging treatment landscape of mCRC.

## Conclusion

Our study discovered a new biomarker, the STAT1, for immunotherapy on mCRC treatment. We also provided a potential novel rationale for the addition of bortezomib, a proteasome inhibitor, in new immunotherapy combinations for negative STAT1 mCRC.

## Supplementary Information

**Additional file 1:** Raw data of the PerkinElmer Opal multiplex analysis from 60 samples. The analysis was summarized in Table 1.

## Data Availability

We enrolled patients at National Taiwan University Hospital. Detailed information for the processes of PerkinElmer Opal multiplex system is available on the official website at https://www.perkinelmer.com/lab-solutions/resources/docs/DTS_1-05-40-NR-OPALGUIDELINES_Opal4-7-color_Manual_Kit_Insert.pdf. Quantification of the biomarkers in the tissue sections were performed using the Vectra Polaris Automated Quantitative Pathology Imaging System along with inForm analysis software (PerkinElmer, CLS143455) at our lab. Detailed information and the gene list of NanoString are available on the official website at https://www.nanostring.com/products/gene-expression-panels/gene-expression-panels-overview/hallmarks-cancer-gene-expression-panel-collection/pancancer-immune-profiling-panel.

## Abbreviations

MSI-H: Microsatellite instability-high
mCRC: Metastatic colorectal cancer
5-FU: 5-Fluorouracil
IFN-γ: Interferon-γ
JAK: Janus kinase
STAT: Signal transducer and activator of transcription
MHC: Major histocompatibility complex
CRC: Colorectal cancer
PD-L1: Programmed cell death-ligand 1
MSS: Microsatellite stable
LLnL: N-acetyl-L-leucyl-L-leucyl-L-norleucinal
HLA: Human leukocyte antigen
PBS: Phosphate-buffered saline
Ig: Immunoglobulin
PE: Phycoerythrin
pSTAT1: Phosphorylated STAT 1
HRP: Horseradish peroxidase
SDS-PAGE: Sodium dodecyl sulfate polyacrylamide gel electrophoresis
DAPI: 4′,6-Diamidino-2-phenylindole
IHC: Immunohistochemical
pan-CK: Pan-cytokeratin
IRF-1: Interferon regulatory factor-1
TILs: Tumor infiltrating lymphocytes
mRNA: Messenger ribonucleic acid
